# Proactive Prophylaxis With Azithromycin and HydroxyChloroquine in Hospitalised Patients With COVID-19 (ProPAC-COVID): A structured summary of a study protocol for a randomised controlled trial

**DOI:** 10.1186/s13063-020-04409-9

**Published:** 2020-06-10

**Authors:** Pradeesh Sivapalan, Charlotte Suppli Ulrik, Rasmus Dahlin Bojesen, Therese Sophie Lapperre, Josefin Viktoria Eklöf, Kjell Erik Julius Håkansson, Andrea Browatzki, Casper Tidemansen, Jon Torgny Wilcke, Julie Janner, Vibeke Gottlieb, Howraman Meteran, Celeste Porsbjerg, Birgitte Lindegaard Madsen, Mia Moberg, Lars Pedersen, Thomas Lars Benfield, Jens Dilling Lundgren, Filip Krag Knop, Tor Biering-Sørensen, Muzhda Ghanizada, Tine Peick Sonne, Uffe Christian Steinholtz Bødtger, Sidse Graff Jensen, Daniel Bech Rasmussen, Eva Brøndum, Oliver Djurhuus Tupper, Susanne Wiemann Sørensen, Gitte Alstrup, Christian Borbjerg Laursen, Ulla Weinrich Møller, Asger Sverrild, Jens-Ulrik Stæhr Jensen

**Affiliations:** 1 Section of Respiratory Medicine, Department of Medicine, Herlev and Gentofte Hospital University of Copenhagen, Hellerup, Denmark; 2 Department of Internal Medicine, Zealand University Hospital, Roskilde, Denmark; 3 Department of Respiratory Medicine, Amager and Hvidovre Hospital University of Copenhagen, Hvidovre, Denmark; 4 Department of Surgery, Næstved-Slagelse- Ringsted Hospitals University of Southern Denmark, Slagelse, Denmark; 5 Department of Respiratory Medicine, Bispebjerg and Frederiksberg Hospital University of Copenhagen, Copenhagen, Denmark; 6 Department of Respiratory and Infectious Diseases, Nordsjællands Hospital University of Copenhagen, Hillerød, Denmark; 7 Dep. of Infectious Diseases, Amager and Hvidovre Hospital University of Copenhagen, Hvidovre, Denmark; 8 Department of Infectious Diseases, Rigshospitalet University of Copenhagen, Copenhagen, Denmark; 9 Department of Clinical Metabolic Research, Herlev and Gentofte Hospital University of Copenhagen, Hellerup, Denmark; 10 Department of Cardiology, Herlev and Gentofte Hospital University of Copenhagen, Hellerup, Denmark; 11 Department of Respiratory Medicine, Næstved-Slagelse-Ringsted Hospitals University of Southern Denmark, Slagelse, Denmark; 12 Institute for Regional Health Research University of Southern Denmark, Odense, Denmark; 13 Department of Respiratory Medicine, Odense University Hospital University of Southern Denmark, Odense, Denmark; 14 Department of Respiratory Medicine, Aalborg University Hospital University of Aalborg, Aalborg, Denmark

**Keywords:** COVID-19, Randomised controlled trial, protocol, azithromycin, hydroxychloroquine, respiratory infections

## Abstract

**Objectives:**

The aim of this randomised GCP-controlled trial is to clarify whether combination therapy with the antibiotic azithromycin and hydroxychloroquine via anti-inflammation/immune modulation, antiviral efficacy and pre-emptive treatment of supra-infections can shorten hospitalisation duration for patients with COVID-19 (measured as "days alive and out of hospital" as the primary outcome), reduce the risk of non- invasive ventilation, treatment in the intensive care unit and death.

**Trial design:**

This is a multi-centre**,** randomised, Placebo-controlled, 2-arm ratio 1:1, parallel group double-blind study.

**Participants:**

226 participants are recruited at the trial sites/hospitals, where the study will take place in Denmark: Aalborg, Bispebjerg, Gentofte, Herlev, Hillerød, Hvidovre, Odense and Slagelse hospitals.

Inclusion criteria:

• Patient admitted to Danish emergency departments, respiratory medicine departments or internal medicine departments

• Age≥ 18 years

• Hospitalized ≤48 hours

• Positive COVID-19 test / diagnosis during the hospitalization (confirmed).

• Men or non-fertile women. Fertile women* must not be pregnant, i.e. negative pregnancy test must be available at inclusion

• Informed consent signed by the patient

*Defined as after menarche and until postmenopausal (no menstruation for 12 months)

Exclusion criteria:

• At the time of recruitment, the patient uses >5 LO2/min (equivalent to 40% FiO2 if measured)

• Known intolerance/allergy to azithromycin or hydroxychloroquine or hypersensitivity to quinine or 4-aminoquinoline derivatives

• Neurogenic hearing loss

• Psoriasis

• Retinopathy

• Maculopathy

• Visual field changes

• Breastfeeding

• Severe liver diseases other than amoebiasis (INR> 1.5 spontaneously)

• Severe gastrointestinal, neurological and hematological disorders (investigator-assessed)

• eGFR <45 ml/min/1.73 m2

• Clinically significant cardiac conduction disorders/arrhythmias or prolonged QTc interval (QTc (f) of> 480/470 ms).

• Myasthenia gravis

• Treatment with digoxin*

• Glucose-6-phosphate dehydrogenase deficiency

• Porphyria

• Hypoglycaemia (Blood glucose at any time since hospitalization of <3.0 mmol/L)

• Severe mental illness which significantly impedes cooperation

• Severe linguistic problems that significantly hinder cooperation

• Treatment with ergot alkaloids

*The patient must not be treated with digoxin for the duration of the intervention. For atrial fibrillation/flutter, select according to the Cardiovascular National Treatment Guide (NBV): Calcium antagonist, Beta blocker, direct current (DC) conversion or amiodarone. In case of urgent need for digoxin treatment (contraindication for the aforementioned equal alternatives), the test drug should be paused, and ECG should be taken daily.

**Intervention and comparator:**

Control group:

The control group will receive the standard treatment + placebo for both types of intervention medication at all times. If part or all the intervention therapy being investigated becomes standard treatment during the study, this may also be offered to the control group.

Intervention group:

The patients in the intervention group will also receive standard care. Immediately after randomisation to the intervention group, the patient will begin treatment with:

Azithromycin:

Day 1-3: 500 mg x 1

Day 4-15: 250 mg x 1

If the patient is unable to take the medication orally by themselves, the medication will, if possible, be administered by either stomach-feeding tube, or alternatively, temporary be changed to clarithromycin 500 mg x 2 (this only in agreement with either study coordinator Pradeesh Sivapalan or principal investigator Jens-Ulrik Stæhr Jensen). This will also be done in the control group if necessary. The patient will switch back to azithromycin when possible.

Hydroxychloroquine:

Furthermore, the patient will be treated with hydroxychloroquine as follows:

Day 1-15: 200 mg x 2

**Main outcomes:**

• Number of days alive and discharged from hospital within 14 days (summarises both whether the patient is alive and discharged from hospital) ("Days alive and out of hospital")

**Randomisation:**

The sponsor (Chronic Obstructive Pulmonary Disease Trial Network, COP:TRIN) generates a randomisation sequence. Randomisation will be in blocks of unknown size and the final allocation will be via an encrypted website (REDCap). There will be stratification for age (>70 years vs. <=70 years), site of recruitment and whether the patient has any of the following chronic lung diseases: COPD, asthma, bronchiectasis, interstitial lung disease (Yes vs. No).

**Blinding (masking):**

Participants and study personnel will both be blinded, i.e. neither will know which group the participant is allocated to.

**Numbers to be randomised (sample size):**

This study requires 226 patients randomised 1:1 with 113 in each group.

**Trial Status:**

Protocol version 1.8, from April 16, 2020. Recruitment is ongoing (first patient recruited April 6, 2020; final patient expected to be recruited October 31, 2020).

**Trial registration:**

ClinicalTrials.gov Identifier: NCT04322396 (registered March 26, 2020)

**Full protocol:**

The full protocol is attached as an additional file, accessible from the Trials website (Additional file 1). In the interest in expediting dissemination of this material, the familiar formatting has been eliminated; this Letter serves as a summary of the key elements of the full protocol.

The study protocol has been reported in accordance with the Standard Protocol Items: Recommendations for Clinical Interventional Trials (SPIRIT) guidelines (Additional file 2).

## Supplementary information

**Additional file 1.** Full study protocol.

**Additional file 2.** SPIRIT checklist.

## Data Availability

It is the opinion of the COP:TRIN steering committee that knowledge sharing creates more and better scientific results. Requests for trial information can be submitted to the Project Management (Jens-Ulrik Jensen, Charlotte Ulrik, Pradeesh Sivapalan) who will consider the request. Any reasonable requests will then be discussed with the COP:TRIN Steering Committee.

