# Supplementary material for: Proactive Prophylaxis With Azithromycin and HydroxyChloroquine in Hospitalised Patients With COVID-19 (ProPAC-COVID): A structured summary of a study protocol for a randomised controlled trial
Source: Trials. 2020 Jun 10;21:513. doi: 10.1186/s13063-020-04409-9 (PMC7284668; doi:10.1186/s13063-020-04409-9)
Supplement: Supplementary file 1 — Additional file 1. Full study protocol. [file 13063_2020_4409_MOESM1_ESM.pdf]

16APR2020 **V.1.8**

Study Protocol - Proactive Prophylaxis With Azithromycin and Chloroquine in Hospitalised Patients With COVID-19 (ProPAC-COVID)

A Randomised, Good-Clinical-Practice-monitored, Placebo-controlled, double-blinded study to clarify whether hospital length and risk of intensive care stay may be reduced in hospitalized patients who have COVID-19 treated with azithromycin and hydroxychloroquine for 15 days after inclusion.

Organisation: COP:TRIN – Chronic Obstructive Pulmonary Disease Trial Network: www.coptrin.dk

Authors:

Pradeesh Sivapalan[1,2], Charlotte Suppli Ulrik[3], Rasmus Dahlin Bojesen[4], Therese Sophie Lapperre[5], Josefin Viktoria Eklöf[1], Kjell Erik Julius Håkansson[3], Andrea Browatzki[6], Casper Tidemandsen[3], Jon Torgny Wilcke[1], Julie Janner[3], Vibeke Gottlieb[1], Howraman Meteran[1], Celeste Porsbjerg[5], Birgitte Lindegård Madsen[6], Mia Moberg[3], Lars Pedersen[5], Thomas Lars Benfield[7], Jens Dilling Lundgren[8], Filip Krag Knop[9], Tor Biering-Sørensen[10], Muzhda Ghanizada[5], Tine Peick Sonne[1], Uffe Christian Steinholtz Bødtger[2, 11, 12], Sidse Graff Jensen[1], Daniel Bech Rasmussen[11], Eva Brøndum[3], Oliver Djurhuus Tupper[3], Susanne Wiemann Sørensen[3], Gitte Alstrup[11], Christian Borbjerg Laursen[13], Ulla Møller Weinreich[14], Asger Sverrild[5], Jens-Ulrik Stæhr Jensen[1] (corresponding)

[1]Section of Respiratory Medicine, Dep. of Medicine, Herlev and Gentofte Hospital University of Copenhagen, Hellerup, DK
[2]Dep. of Internal Medicine, Zealand University Hospital, Roskilde, DK
[3]Dep. of Respiratory Medicine, Amager and Hvidovre Hospital University of Copenhagen, Hvidovre, DK
[4]Dep. of Surgery, Næstved-Slagelse-Ringsted Hospitals University of Southern Denmark, Slagelse, DK
[5]Dep. of Respiratory Medicine, Bispebjerg and Frederiksberg Hospital University of Copenhagen, Copenhagen, DK
[6]Dep. of Respiratory and Infectious Diseases, Nordsjællands Hospital University of Copenhagen, Hillerød, DK
[7]Dep. of Infectious Diseases, Amager and Hvidovre Hospital University of Copenhagen, Hvidovre, DK
[8]Dep. of Infectious Diseases, Rigshospitalet University of Copenhagen, Copenhagen, DK
[9]Dep. of Clinical Metabolic Research, Herlev and Gentofte Hospital University of Copenhagen, Hellerup, DK
[10]Dep. of Cardiology, Herlev and Gentofte Hospital University of Copenhagen, Hellerup, DK
[11]Dep. of Respiratory Medicine, Næstved-Slagelse-Ringsted Hospitals University of Southern Denmark, Slagelse, DK
[12]Institute for Regional Health Research University of Southern Denmark, Odense, DK
[13]Dep. of Respiratory Medicine, Odense University Hospital University of Southern Denmark, Odense, DK
[14]Dep. of Respiratory Medicine, Aalborg University Hospital University of Aalborg, Aalborg, DK

This study is part of the Danish national non-commercial lung research network COP:TRIN (www.coptrin.dk) and is conducted as a randomised controlled trial.

Hypothesis:

→ In patients with urgent hospital admission and a positive test SARS-CoV-2 treatment with hydroxychloroquine azithromycin leads to shorter hospitalisation and fewer admissions to the intensive care unit.

EUdraCT-no.: 2020-001198-55
ClinicalTrials.gov Identifier: NCT04322396
The Danish Medicines Agency case no.: 2020033414
The Danish National Committee on Health Research Ethics approval no.: H-20022574
The Danish Data Protection Agency journal no.: P-2020-258

Principal investigator / Study director and scientific sponsor:
Research Associate Professor, Research Manager, Jens-Ulrik Stæhr Jensen, MD, PhD.
Research Unit, Pulmonary Medicine Section, Gentofte Hospital, Kildegårdsvej 28, 2900 Hellerup,
Tel: 38 67 30 57 + 28 93 81 68. Email: jens.ulrik.jensen@regionh.dk

Research Centres:

1. Pulmonary medicine section, Gentofte hospital, Kildegårdsvej 28, 2900 Hellerup

Primary investigator: Jens-Ulrik Stæhr Jensen.

Other investigators: Associate Professor, Senior Consultant Saher Shaker, Associate Professor, Senior Consultant Torgny Wilcke, Senior Consultant Karin Armbruster, Postdoc Pradeesh Sivapalan MD, PhD, PhD student Josefin Eklöf, MD, Howraman Meteran, MD, PhD, Chief physician Mette Vang Larsen, Chief physician Sidse Graff Jensen, PhD.

2. Pulmonary Medicine Department, Hvidovre Hospital, Kettegaard alle 30, Hvidovre
Primary Investigator: Professor Charlotte Suppli Ulrik
Other investigators: Chief physician Julie Janner, Mia Moberg, MD, PhD, PhD student Kjell Håkansson, MD.

3. Pulmonary Medicine Section, Herlev Hospital, Herlev Ringvej, Herlev
Primary Investigator: Professor Peter Lange
Other investigators: Hans Henrik Lawaetz-Schultz, MD, PhD, Chief physician Vibeke Gottlieb, Chief physician, Christian Søborg, PhD, Chief Physician Pernille Ravn, PhD.

4. Pulmonary and infectious medicine department, North Zealand Hospital, Dyrehavevej 29, 3400 Hillerød
Primary Investigator: Chief physician Andrea Browatzki
Other investigators: Chief physician Birgitte Lindegaard Madsen, DMSc, Chief physician Zitta Barrella Harboe, PhD, Senior Consultant Thyge Lynghøj Nielsen, PhD.

5. Pulmonary Medicine Department, Aalborg Hospital, Hobrovej 18 -22, 9000 Aalborg
Primary investigator: Associate Professor, consultant, PhD, Ulla Møller Weinreich
Other investigators: PhD student Peter Ascanius Jakobsen, PhD student Mia Solholt Godthaab Brath.

6. Pulmonary Medicine Section, Medical Department, Roskilde Hospital, Roskilde
Primary investigator: Chief physician, PhD, Christian Niels Meyer
Coordinating Investigator: post doc, , Pradeesh Sivapalan

7. Pulmonary Medicine Department, Bispebjerg Hospital, Bispebjerg Bakke 23, 2400 Copenhagen
Primary investigator: Research Associate Professor, Chief Physician, PhD, Therese Lapperre

8. Medical Department,Næstved-Slagelse-Ringsted Hospital
Primary investigator: Consultant in Pulmonology, PhD, associate research professor (University of Southern Denmark)Uffe Bødtger.

9. Medical department, Odense University Hospital Primary investigator: Research Associate Professor, Chief Physician, PhD. Christian B. Laursen Investigator: Professor, Chief Physician, DMSc, Isik Somuncu Johansen

Other participants (not experimental centres)

1. Infectious Medicine Department, Rigshospitalet, Blegdamsvej, Copenhagen Ø.
Primary Investigator: Professor Jens D. Lundgren

2. Intensive Treatment Section 41.31, Rigshospitalet, Blegdamsvej, Copenhagen Ø. Professor, Chief Physician, Anders Perner, DMSc

3. Center for Rheumatology and Spine diseases, Rigshospitalet, Blegdamsvej, Copenhagen Ø.
Professor, Chief Physician, Søren Jacobsen, DMSc

4. Intensive care unit, Gentofte Hospital, Niels Andersen road 65, 2900 Hellerup.
Head of Section, Thomas Mohr, Ph.D.

5. Intensive therapy section, NOH, Hillerød, Helsevej 2, 3400 Hillerød Research Associate Professor, Morten Bestle, Ph.D.

6. Cardiology department, Herlev-Gentofte Hospital, Niels Andersens Vej 65, 2900 Hellerup
Associate Professor Tor Biering-Sørensen Ph.D.

7. Clinical pharmacology department, Bispebjerg Hospital, Bispebjerg Bakke 23, 2400 Copenhagen
Associate Professor, Doctor, Ph.D. Tonny Studsgaard Petersen

8. Cardiac Catherization Laboratory 20.14, Heart Center, Rigshospitalet, Blegdamsvej, Copenhagen Ø Professor, Jens Kastrup, DMSc

GCP monitoring:

The GCP units by GCP coordinator Kristina Devantier

COP:TRIN, Steering Committee: Please see: http://coptrin.dk/steering-committee-members/

Background information on the medication used in this trial:

Azithromycin

Categorized as an antibacterial agent. The drug is an approved, and marketed in Denmark, for treatment of upper and lower respiratory tract infection. Azithromycin is also, according to available evidence and current guidelines, used for treatment of asthma, COPD and bronchiectasis in order to improve disease control and reduce exacerbation rate. Please see enclosed product summaries for further information.

Hydroxychloroquine:

Categorized as an anti-inflammatory and anti-malaria agent.

The drug is approved and marketed in Denmark for the prevention and treatment of malaria, for the treatment of rheumatoid arthritis, discoid and systemic lupus erythematosus and juvenile idiopathic arthritis.

Manufacturing, packaging and labelling of IMP:

The trial drugs (IMP) are manufactured by Glostrup pharmacy by pharmacist Kristian Østergaard Nielsen. Placebo capsules are thus made similar to the intervention medicine. The drug (and Placebo) are labelled, according to Appendix 13. Glostrup pharmacy has a key for blinding. Therefore, it will always be possible to unblind a patient if indicated. Investigator must notify sponsor on grounds if a patient is unblinded.

Medical professionals dispense the IMP daily during hospitalization, except for patients preferring to handle their medication themselves. In the latter case, patient will fill in a medication diary (provided at randomisation). For all patients who are discharged during the intervention period, a medication diary will be provided. The medication diary is subsequently collected.

Exactly the amount of medication or placebo that the patient is required to take during the study periode will be provided, but for patients who do not take all the medication (protocol deviation), the remaining medication will be collected by the study staff. The patient will receive a follow-up phone call to check whether they have adhered to the medication schedule according to the trial protocol.

1. Hypothesis and aims:

1.1 Hypothesis:

In patients with acute hospital admission, a positive test for 2019-nCoV and symptoms of COVID-19 disease, treatment with virus-modifying agent hydroxychloroquine as well as virus-immunomodulatory and antibacterial drug azithromycin can lead to shorter hospitalisation and fewer admissions to the intensive care unit.

1.2 Aims:

The aim of this randomised GCP-controlled trial is to clarify whether combination therapy with macrolide azithromycin and hydroxychloroquine via anti-inflammation/immune modulation, antiviral efficacy and pre-emptive treatment of supra-infections can shorten hospitalisation duration (measured as "days alive and out of hospital"as the primary outcome), reduce the risk of non-invasive ventilation, treatment in the intensive care unit and death.

2. Background and scientific perspective:

2.1 Background - rationale

Coronavirus - COVID-19

In the ongoing coronavirus pandemic, COVID-19, with its origin in Wuhan, China, there is still sparse data on the course, risk of various complications, and the best possible treatment of patients admitted to hospital to ensure best possible survival and reducing length of stay at hospital. The most frequent symptoms are fever (> 80%) and cough (70-80%), together with radiologically fingdings of "ground-glass infiltrates" or "patchy infiltrates" in the patients with the most severy ill patients (86%), compatible with severe viral pneumonitis (1, 2). The length of hospitalisation is observed to be relatively long, 10-15 days (3), which in itself is a problem as hospitals can quickly reach the maximum capacity for hospitalisation and the proportion of patients who become critically ill have, based on the observations reported so far, had a mortality rate of> 60% (4), and overall mortality for admitted patients in China with COVID-19 infection is apparently unusually high for viral respiratory tract infections with an estimate of 25% (2).

COVID-19 and lung disease

Only specific data on patients with Chronic Obstructive Pulmonary Disease (COPD) have been reported in a few studies, but the risk of in-hospital death appears to be very high (OR 5.4 [95% CI 0.96-30.40]) (2).

Lack of specific treatment

Despite the rapid spread of the disease globally, there is no solid data yet to recommend any specific treatments, and by that, symptomatic, organ supportive therapy is recommended, and in case of progression to severe acute respiratory failure mechanical ventilation (5). A high incidence of bacterial super-infections has been reported in patients with COVID-19 who died (50%) compared to survivors (1%) (p <0.0001), and likewise, an incidence of septic shock of 70% and 0%, respectively (2). Thus, there is an urgent need for treatments that can improve the course of the diseases in the individual patients, including positive impact on risk for hospital admission, duration of hospitalisation, risk of secondary infections and death.

Macrolide azithromycin as a possible treatment for patients with COVID-19

Azithromycin is a macrolide antibiotic that has shown convincing efficacy in several studies in recent years to reduce hospitalisation-related exacerbations in COPD (6, 7), and to reduce exacerbationrate in asthma (8) and non-cystic fibrosis bronchiectasis (9). At the same time, it has been shown that azithromycin has a distinct effect by down-regulating airway inflammation by reducing CXCL1, TNF-alpha, IL-13 and IL-12p40 (10). Furthermore, a strong association has also been reported between survival from Acute Respiratory Distress Syndrome (ARDS) and administration of azithromycin (HR for 90 days of death for all causes: 0.49 [95% CI 0.27 - 0.87] in a well-conducted study (11).

Furthermore, it has been consistently observed in several recent publications that azithromycin itself appears to have an antiviral effect on a number of several viruses causing respiratory tract infections, such as Respiratory Syncytial Virus (RSV) (12), Rhinovirus (13) and Zika virus (14).

Hydroxychloroquine as immunomodulatory and antiviral agent by COVID-19

Hydroxychloroquine has been marketed since 1934, originally developed for prophylaxis and treatment of malaria, but has for years also been used an anti-inflammatory agent for rheumatic diseases. Large daily doses (up to 400 mg a day) of hydroxychloroquine are prescribed over many years to patients with arthritis such as systemic lupus erythematosus and rheumatoid arthritis for anti-inflammatory purposes, which is generally well-tolerated (16). but in addition to these effects, it is well described that the drug has an antiviral effect especially against flavivirus, retrovirus and coronavirus by inhibiting a number of low-pH-dependent steps in virus replication, as well as by inhibiting the pH-dependent endosomal mediated viral uptake in cells (15). The drug is well tolerated even with high dosage, for up to five years and there is no signal for birth defects with usage of the drug summarised by Savarino et al. (15).

Cell studies with primate cells infected with the coronavirus that induced SARS-1 (formerly called SARS) have shown that chloroquine, in a dose-dependent manner, inhibits the ability of the corona virus to infect cells and to spread among cells (17). Thus, several researchers and health care professionals have, during the present SARS-CoV-2 pandemic, have proposed studies examining hydroxychloroquine/chloroquine as treatment for patients with COVID-19 disease (18, 19).

2.2 Scientific perspective for this study

The study will clarify whether treatment with azithromycin in combination with hydroxychloroquine for 15 days from the time of hospital admission with diagnosed COVID-19 disease may reduce the length of hospitalisation, the risk of admission to the intensive care unit, treatment with non-invasive ventilation and death. The study will also clarify whether this treatment can reduce the need for oxygen supplementation (time for breathing on its own without oxygen supplementation) or for regular Long-Term Oxygen Therapy oxygen supplementation ("home oxygen").

If the treatment also improves the course of COVID-19 disease in patients with pre-existing lung disease, a very large number of patients could benefit from the treatment immediately.

The study originates from the Danish national non-commercial lung research network COP:TRIN (Chronic Obstructive Pulmonary Disease: Trial Network).

3. Method:

3.1 Design:

Randomised, Good-Clinical-Practice-monitored, placebo-controlled, double-blind study.

3.2. Recruitment and inclusion:

See point 12.

3.3 Inclusion:

<u>Inclusion criteria:</u>

• Patient admitted to Danish emergency departments, respiratory medicine departments or internal medicine departments
• Age≥ 18 years
• Hospitalized ≤48 hours
• Positive SARS-CoV-2 test / diagnosis during the hospitalization (confirmed).
• Men or non-fertile women. Fertile women* must not be pregnant, i.e. negative pregnancy test must be available at inclusion
• Informed consent signed
*Defined as after menarche and until postmenopausal (no menstruation for 12 months)

<u>Exclusion criteria:</u>

• At the time of recruitment, the patient uses >5 LO2/min (equivalent to 40% FiO2 if measured)
• Known intolerance/allergy to azithromycin or hydroxychloroquine or hypersensitivity to quinine or 4-aminoquinoline derivatives

• Neurogenic hearing loss
• Psoriasis
• Retinopathy
• Maculopathy
• Visual field changes
• Breastfeeding
• Severe liver diseases other than amoebiasis (spontaneous INR > 1.5)
• Severe gastrointestinal, neurological and hematological disorders (investigator-assessed)
• eGFR <45 ml/min/1.73 m2
• Clinically significant cardiac conduction disorders/arrhythmias or prolonged QTc interval (QTc (f) of> 480/470 ms).
• Myasthenia gravis
• Treatment with digoxin*
• Glucose-6-phosphate dehydrogenase deficiency
• Porphyria
• Hypoglycaemia (Blood glucose at any time since hospitalization of <3.0 mmol/L)
• Severe mental illness which significantly impedes cooperation
• Severe linguistic problems that significantly hinder cooperation
• Treatment with ergot alkaloids
*The patient must not be treated with digoxin for the duration of the intervention. For atrial fibrillation/flutter, select according to the Cardiovascular National Treatment Guide (NBV): Calcium antagonist, Beta blocker, direct current (DC) conversion or amiodarone. In case of urgent need for digoxin treatment (contraindication for the aforementioned equal alternatives), the test drug should be paused, and ECG should be recorded daily.

Drug Interactions

Interactions should be taken into account if the patient is taking other medications. For azithromycin, this includes antacids, ergotamine derivatives, colchicine and cyclosporine. For hydroxychloroquine, these include antidiabetic agents, tricyclic antidepressants, antipsychotics, halofantrine, cyclosporine, mefloquine, antiepileptic drugs, praziquantel and agalsidase.

Standard Treatment

As there is no specific treatment for COVID-19, standard assessment and treatment is based on organ supportive therapy such as oxygen therapy (central), fluid therapy, antibiotic therapy for secondary infections. If the disease progress to severe acute respiratory failure, the patients will often require referral to an intensive care unit for mechanical ventilation.

In addition, Danish national guidelines for handling of in-hospital Covid-19 patients can be obtained from www.lungemedicin.dk or from the open access journal European Respiratory Clinical Journal (at present in press).

3.3.2. Allocation:

The sponsor generates a randomisation sequence. Randomisation will be in blocks of unknown size and the final allocation will be via an encrypted website (REDCap), where also inclusion and exclusion criteria are required to be filled in correctly in order to randomise a patient.

Allocation will be stratified for age (>70 years vs. <=70 years), site of recruitment and whether the patient has any of the following chronic lung diseases: COPD, asthma, bronchiectasis, interstitial lung disease (Yes vs. No).

3.3.3. Data collection, surveys and follow-up:

The primary daily project management is carried out by the project manager. In addition, a project group (investigators), consisting of doctors from the departments involved, is trained to assist the project manager with the recruitment, sampling and follow-up of patients. All medical decisions regarding patients will be taken by a physician. Data is collected in Case Report Forms (CRF) for each individual patient.

Prior to consent to participate in the trial, we will only assess the specific information needed to assess inclusion and exclusion criteria. No other information will be accessed.

It is the attending physician who asks if patients are interested in hearing more about the trial. If yes, an investigator is contacted, who will inform the patient about the trial.

As part of the study, all patients will be regularly monitored for oxygen saturation, heart rate, blood pressure, respiratory rate and temperature during hospitalisation.

The following information will also be obtained:
• Date of birth, age and gender
• Height, weight and BMI
• Use of medicine
• For patients with chronic obstructive pulmonary disease (COPD) classification in GOLD 1-4 and GOLD A-D (symptoms assessed via MRC degree)
• Systematic screening for co-morbidities
• Smoking history with pack years, current smoking status and alcohol consumption
• Information from the chest x-ray
• Arterial-blood gas analysis (pH, pCO2, pO2, Base Excess, oxygen supplementation)
• Hb, leucocytes + differential count, CRP, kidney function, liver parameters, electrolytes, LDH
• ECG, vital status, and adverse events

And from the patients' medical journal:

- CT thorax
- Home oxygen supplementation and dosage
- Home NIV, acute NIV and respirator therapy
- Re-admission
- Prescribed medication

All this information is passed on to the investigator. The information from the medical records is required to calculate demographic data, medication data and outcomes in the trial. No information that is not required according to the protocol will be obtained. Case Report Form is archived at the departments involved for 15 years. A separate database is created in REDCap (www.projectredcap.org) for data management.

Table 1 (SPIRIT Figure): Overview of examinations that each participant will undergo:

	STUDY PERIOD					
	Enrolment	Allocation	Post-allocation (days)			
Timepoint	-24 hours	1	2-3	4	5 to discharge	15
ENROLMENT:						
Informed consent	X					
Eligibility screening	X					
Allocation		X				
INTERVENTION:						
Group 0: Placebo treatment		←	—	—	—	→
Group 1: Azithromycin /hydroxychloroquine treatment		←	—	—	—	→
EXAMINATIONS:						
Blood sample*		X	X	X	X	
Pregnancy test (only fertile women)		X				
Arterial blood gas		X		X		
ECG**		X	X***	X	X	
MRC score + GOLD****		X				
Body mass index (BMI)		X				
Oxygen supplementation (L/min) (open)		X	X	X	X	
FiO2 (%) (respirator)		N/A	X	X	X	
Chest x-ray		During hospital admission				

*The blood samples include haemoglobin (Hb), leukocytes + differential count, thrombocytes, C-reactive protein (CRP), Na+, K+, albumin, creatinine, urea, amylase, alkalic phosphatase, beta-2-Microglubulin, fibrinogen, glucose, TSH, INR, billirubin, D-dimer, APTT, calcium, triglycerides, ferritin and lactate dehydrogenase (LDH). These blood tests will also be recommended daily for COVID patients outside studies in the recommendation of the Danish lung medicine association. **When screening for the study, any ECG from within the last 3 days can be used. ***A follow-up ECG can be recorded during any remaining days of the hospital admission. ****Only in patients with COPD.

3.3.4 Intervention

Neither patients nor study staff will know which group the patient is allocated to. The medicine will be marked neutral, e.g. "Azithromycin group A" and "Azithromycin group B" and the same for Hydroxychloroquine.

NOTE: If the patient is receiving azithromycin prophylaxis, common practice is followed: the prophylaxis is paused and then restarted as usual.

Control group:

The control group will receive the standard treatment + placebo for both types of intervention IMP at all times. If part or all the intervention therapy being investigated becomes standard treatment during the study, this may also be offered to the control group.

Intervention group:

The patients in the intervention group will also receive standard care. Immediately after randomisation to the intervention group, the patient will begin treatment with :

Azithromycin:

Day 1-3: 500 mg x 1

Day 4-15: 250 mg x 1

If the patient is unable to take the medication orally by themselves, the medication will, if possible, be administered by either stomach-probe, or alternatively, temporary be changed to clarithromycin 500 mg x 2 (this only in agreement with either study coordinator Pradeesh Sivapalan or principal investigator Jens-Ulrik Stæhr Jensen). This will also be done in the control group if necessary. The patient will switch back to azithromycin when possible.

Hydroxychloroquine:

Furthermore, the patient will be treated with hydroxychloroquine as follows:

Day 1-15: 200 mg x 2

Follow-up is done on days 14, 29, 90 and 365 days, by accessing the electronic medical record system. The specific information obtained, and its purpose can be found in section 3.3.3.

Regarding dosage

The Summary of Product Characteristics for Azithromycin suggests 500mg/day for three days or 500mg/day for one day and then 250mg daily for four days. However, other clinical studies have found a positive effect of a daily dose of 250 mg for prolonged periods as prophylactic treatment. Mortality among hospitalised patients with COVID-19 is quite high and the median time for hospitalisation is 10-15 days, so it seems reasonable to give patients prophylactic dose for 12 days.

The dosing of hydroxychloroquine follows the summary of product characteristics.

Regarding other treatment with antibiotics:

If antibiotic therapy is deemed indicated to the patient due to e.g. pneumonia or if it becomes standard therapy, piperacillin-tazobactam should be given as an empirical treatment at a dose adjusted to renal function. In case of penicillin allergy, cefuroxime is also given at a dose appropriate to renal function, weight and age. When positive microbiology is available, immediately switch to targeted treatment.

If *specific suspicion of atypical pneumonia* is raised, ciprofloxacin is administered at a dose of corresponding to kidney function and concomitant examination for atypical pneumonia will be performed. If negative, ciprofloxacin is discontinued. If positive, ciprofloxacin treatment is continued for the duration of treatment corresponding to the microorganism detected. If there is a specific need for treatment with macrolide and where other options are not available (e.g. allergy to fluoroquinolones, or when there is an estimated need for combination treatment of e.g. legionella pneumonia), consult with an investigator, and in this case it may be decided to discontinue azithromycin (active) or azithromycin placebo. In this case, treatment stops without unblinding.

Furthermore, ECG recordings during the treatment period will be analysed with focus on QTc. At QTc (F)> 480/470 ms for respectively women and men, IMP will be discontinued for safety reasons (but the patient remains in the study).

3.3.5. Statistical analyses:

<u>Primary endpoint:</u>

• Number of days alive and discharged from hospital within 14 days (summarises both whether the patient is alive and discharged from hospital) ("Days alive and out of hospital")

<u>Secondary endpoints:</u>

Secondary endpoint no. 1:

Ordinate outcome. The patient is categorized into one of the following 8 categories on day 15:

1. Dead
2. Hospitalised and mechanical ventilation or ExtraCorporalMembraneOxygenation (ECMO)
3. Hospitalised and Non-invasive ventilation or high-flow oxygen device
4. Hospitalised and given oxygen supplements that do not live up to oxygen supplements in (2) or (3) - e.g. oxygen on nasal catheter
5. Hospitalised and do not receive oxygen supplementation but need treatment (COVID-19 related or other)
6. Hospitalised and do not receive oxygen supplements and do not need treatment (just observed)
7. Discharged with restriction on activities, may be free of oxygen depletion or use LTOT ("home oxygen")
8. Discharged, no restrictions on activities

Other secondary endpoints:

• Admitted to the intensive care unit in the two groups (0 vs. 1) during the index admission

  o For patients admitted to intensive care unit: number of days on intensive care (Length of stay, ICU)

• Have been on Non-Invasive Ventilation (NIV) (0 vs. 1) during index hospitalisation

• Dead at day 30

• Duration (days) of index hospital admission

• Days alive and discharged from hospital within 30 days (summarises both whether the patient is alive and discharged from hospital)

• Dead at day 90 (reported later)

• Dead within 12 months (reported later)

• Number of readmissions for all causes within 30 days

• Number of days on NIV or mechanical ventilation during index admission

• Delta PaO_2 day 1 (baseline) to day 4 (72 hours). At the same time oxygen supplements and oxygen systems are registered

• Delta $PaCO_2$ day 1 (baseline) to day 4 (72 hours)

• pH day 4 (72 hours)

• Time to no oxygen supplement (or regular LTOT oxygen supplement)

In addition, several explorative endpoints.

Data is processed and analysed in SAS v.9.4 and graphs are generated in Microsoft Excel and SigmaPlot.

3.3.6. Sample size:

Randomised controlled study

Prerequisite: Type 1 error rate = 5%. Power = 80%. Two-sided statistics. Group sequential design with one scheduled interim analysis after randomisation of 113 patients (50% recruitment).

Analysis: T-test.

Sample size is calculated based on the following estimates and indicative figures:

Expected duration of hospitalisation with COVID-19: 10 days

SD for "Days alive and discharged within 14 days" in patients with lung disease and COVID-19: 4 days (up to 10 days hospitalization)

Estimated time for improvement/deterioration: 1.5 days.

This requires 226 patients randomised 1:1 with 113 in each group. This is a fixed sample size. It is assumed that most patients complete the intervention.

However, for interim analysis, the Data and Safety Monitoring Board (DSMB) may recommend the steering committee to expand sample size.

3.3.7 GCP Monitoring:

Frequency and depth are determined by the GCP units. Initiation visits and the first monitoring visits to all centres will be conducted off-site, i.e. without a physical meeting, due to the SARS-CoV-2 pandemic. Consent sheets will be scanned into an online system (REDCap or journal system) that can be accessed by GCP monitors.

4. Interim Analysis and Data and Safety Monitoring Board

After recruiting half the sample size (approximately 113 patients), an interim analysis will be performed focusing on safety. An external Data and Safety Monitoring Board is appointed.

The interim analysis will be prepared and presented by physician Josefin Eklöf. The groups will be presented as "Group A" and "Group B" and DSMB will only be unblinded if they ask the steering committee for the study on this.

5. Blood samples:

As part of ususal care, blood samples are taken daily from the time of inclusion and as long as the patient is admitted. Blood samples include haemoglobin (Hb), leukocytes + differential count, thrombocytes, C-reactive protein (CRP), Na+, K+, albumin, creatinine, urea, amylase, alkalic phosphatase, beta-2-Microglobulin, fibrinogen, glucose, TSH, D-dimer, APTT, calcium, triglycerides, ferritin, bilirubin, ALAT, INR, and lactate dehydrogenase (LDH), see Table 1. These blood samples are analysed at the hospitals.

In addition, supplemental blood tests and material obtained with nasal swaps will be performed according to the sub-study protocols. Material from this will be included in a research biobank for the trial, and after completion of the trial in another regional biobank. The trial is expected to end in February 2021, and the material will then be transferred to the regional biobank.

For the present trial, the results of blood tests are collected from the patient record.

6. Side effects, risks and disadvantages:

The treating physician may at any time discontinue intervention with IMP if, in clinical and/or paraclinical assessment, it is deemed contraindicated.

Blood tests:

Serious side effects to regular blood sampling (venous puncture) are rare. Frequent (5-15%) can be seen transient discoloration of skin around the insertion site.

X-ray:

Chest X-rays correspond to a radiation dose of approx. 0.1 millisievert (mSv). This should be compared with the average background radiation in Denmark of approx. 3 mSv per year. There are no documented adverse effects of the radiation dose received by Chest X-rays in the literature. Therefore, we believe that the study is not associated with any risks or side effects.

Side effects of the trial medicine:

See www.medicin.dk

Azithromycin:

Very common (>10%) Abdominal pain, Diarrhoea, Flatulence, Nausea.

Common (1-10%) Powerlessness, Decreased appetite. Vomiting, Taste Disorders. Decreased lymphocyte count. Decreased serum bicarbonate. Arthralgia. Headaches, Paraesthesia, Dizziness. Skin itching, rash. Visual disturbances.

Uncommon (0.1-1%) Pain. Hepatitis, Oral candidiasis. Dyspnea, Pneumonia, Oedema. Eosinophilia, Leukopenia, Neutropenia. Elevated serum bicarbonate, Hyperchloremia, Hyperglycaemia, Hyperkalaemia, Hypernatremia, Hypokalaemia, Hyponatraemia. Arthritis, Back Pain. Nervousness, Somnolence. Facial oedema, Dermatitis, Photosensitivity. Candidiasis, Infections. Metrorrhagia, Kidney Pain, Testicular Disease, Vaginitis. Hearing loss, Tinnitus.

Rare (0.01-0.1%) Cholestasis, Liver Impact. Agitation. Acute generalized exanthematous pustulosis*, Allergic reactions*, Angioedema*, Hypersensitivity.

Not known Fulminant hepatitis, Hepatotoxicity, Hepatic insufficiency, Pancreatitis, Pseudomembranous colitis. Arrhythmias, Extended QT interval, Hypotension, Torsades de pointes tachycardia. Haemolytic anaemia, Thrombocytopenia. Aggravated myasthenia gravis. Aggressiveness, Anxiety, Delirium, Hallucinations, Hyperactivity, Hypesthesia, Seizures, Syncope. DRESS - drug reaction with eosinophilia and systemic symptoms, Erythema multiforme, Stevens-Johnson syndrome, Toxic epidermal necrolysis. Anaphylactic reaction. Acute renal failure, Interstitial nephritis.

*In case of allergic reactions, including acute generalized exanthematous pustulosis and DRESS, azithromycin should be discontinued.

<u>Hydroxychloroquine:</u>

Very common (> 10%) Abdominal pain, Nausea.

Common (1-10%) Eating refusals. Diarrhoea, Vomiting. Emotional lability, Headaches. Skin itching, rash. Accommodation difficulty.

Uncommon (0.1-1%) Liver effect. Nervousness, Sensomotor disorders, Dizziness. Alopecia. Corneal oedema, Double vision, Retinopathy *, Tinnitus.

Not known Fulminant liver failure. Bronchospasm, prolonged QT interval**, Cardiomyopathy. Aplastic Anaemia, Bone Marrow Depression, Influence of the Blood Image (agranulocytosis, anaemia, leukocytopenia, thrombocytopenia). Aggravation of Porphyria, Hypoglycaemia. Myopathy. Extrapyramidal genes, Cramps, Neuropathy, Palsy, Psychosis, Suicidal behaviour. Acute generalized exanthematous pustulosis***, Exfoliative dermatitis, Erythema multiforme, Photosensitivity, Stevens-Johnson syndrome, Toxic epidermal necrolysis. Allergic reactions (including DRESS syndrome), Angioedema. Hearing loss, Macular and retinal degeneration, Maculopathy.

*Retinopathy:

- With pigment change requires careful dosing and careful control. When using rheumatologic doses, eye examination by an ophthalmologist is recommended before starting treatment and with follow-up to check for any eye manifestations that may arise. Annual monitoring after 5 years of treatment is recommended, however in risk patients initially annual control, see also Chloroquine derivatives (inflammatory rheumatic diseases), side effects.
- With cardiomyopathy can be fatal.
- With macular degeneration is seen and may be irreversible.
- With reversible corneal changes with oedema and blemishes can cause blurred vision or photophobia.
- With blurred vision accommodation is dose-dependent and reversible.
- In malaria treatment and prophylaxis, fewer and milder side effects occur.

**Prolonged QT interval has been seen in patients with particular risk factors for it.

***Acute generalized exanthematous pustulosis must be distinguished from psoriasis. Psoriasis exacerbation may occur. May be associated with fever and hyperleukocytosis.

An adverse reaction (AR) is defined as any adverse and undesirable reaction to a trial drug regardless of dose. An adverse event (AE) is defined as any adverse event in a patient or subject in a clinical trial following treatment with a drug, without necessarily linking this treatment to the adverse event.

Since the trial drugs are well known and have been used for many years, we will only record side effects not mentioned in the respective drug summary of the trial drug.

A severe adverse reaction or event (SAR/SAE) is defined as an event or adverse event that, regardless of dose, results in death, is life-threatening, results in hospitalization or prolongs hospitalization, results in significant or persistent disability or incapacity, or leading to a congenital anomaly or malformation.

Investigators must immediately (= within 24 hours) report serious incidents and serious adverse reactions (SAEs and SARs) to the sponsor regardless of whether they are described in the respective product summary. This allows the sponsor to assess the benefits and risks along the way in the study.

Events and adverse events recorded during the period from the patient have received the first dose of trial medication up to and including day 15.

Recording and reporting of all events and adverse events will end when the trial drug is stopped.

A high degree of comorbidity and death is seen in this patient group and therefore it is also expected that prolonged admissions, re-admissions, NIV, Respirator treatment and death will occur in this patient group. Therefore, these parameters will not be considered as a SAE.

All incidents and registered side effects are reported at the end of the trial in a final report to the Danish Medicines Agency. All serious suspected adverse reactions must be reported annually together with a report on the safety of the subjects and sent to the the Danish Medicines Agency (LMST) and the Danish National Committee on Health Research Ethics (VEK).

The product summary of the trial drugs is used to assess whether a serious adverse event is unexpected and thus possibly a Suspected Unexpected Serious Adverse Reactions (SUSAR).

In the event of a fatal or life-threatening SUSAR, this must be registered and reported to LMST and VEK within 7 days of the sponsor becoming aware of it. No later than 8 days after the report, the sponsor must provide LMST and VEK with all relevant information about the sponsors and investigators' follow-up on the event. All other SUSARs are reported to LMST and VEK within 15 days of the sponsor becoming aware of them.

The report must be followed up by a detailed written report, and in both the immediate report and the subsequent report, the investigator must identify the subjects with a personal code number. When reporting deaths, the investigator must provide any additional information that the sponsor may request.

7. Economy:

The research project is (investigator) initiated by COP:TRIN. Funding has been obtained from the Novo Nordisk Foundation of DKK 2.2 million for sponsor, remuneration of auxiliary personnel, payment of laboratory tests and equipment, as well as for manufaction of IMP treatment and placebo. The sponsors and investigators are not financially linked to private companies, foundations, etc. in this research project.

Medical expenses are covered, if not obtained from other sources, by the section for respiratory medicine research, Gentofte Hospital.

To the extent possible, the section for respiratory medicine research, Gentofte Hospital supports follow-up for endpoints and otherwise by appointment.

8. Remuneration:

Patients are not paid for participation in the trial.

9. Availability of information and right to data:

The consent gives the primary investigator, monitor and any control authority direct access to obtain information in the patient's medical record, etc., including electronic record, in order to see

information about the subject's health conditions which are necessary as part of the implementation of the research project and for control purposes, including self-monitoring, quality-control etc.

The project group that has designed and conducted this study has the right to data and the right (and duty) to publish based on data. Project management manages data and invites members of the study group to publications. All sites that recruit patients are entitled to at least one authorship on the primary publication, and for every 10 patients recruited, the site is entitled to an extra authorship. Sites that have not participated in the design of the study are entitled to a maximum of 3 authorships. It is the opinion of the steering committee that knowledge sharing creates more and better scientific results. Requests for knowledge sharing from other groups may be submitted to Project Management (Jens-Ulrik Jensen, Charlotte Ulrik, Pradeesh Sivapalan) who will evaluate primarily and who, if the project is found suitable, will discuss it with the COP:TRIN Steering Committee.

Project Management has the first right to undertake sub-studies but may well assign projects to other contributors. In that case, the following considerations will be significant in the assessment: 1) Participation in the design phase of this RCT and at what level, and 2) Number of patients recruited at a site. If the hypothesis to be investigated is not planned to be examined by our group, we will allow the use of our data if the Steering Committee finds the project scientifically sound and, if appropriate, a collaboration with members of the COP:TRIN Steering Committee will be proposed. However, it should be emphasized that data is used for a specific purpose, not for future purposes in general. This becomes conditional by the steering committee for data to be used in a sound way to test hypotheses with relevant scientific content.

Information regarding subjects are processed and stored in accordance with the **Data Protection Regulation (GDPR)**, the Data Protection Act and the Health Act and the project is properly notified in accordance with applicable rules and laws to the appropriate authorities.

10. Publication of results:

All project results will be sought published in scientific contexts, including international journals. This will happen regardless of whether the result is positive, negative or inconclusive.

11. Scientific Ethics Statement:

The study is conducted in accordance with the Declaration of Helsinki and is carried out in accordance with the rules of the Personal Data Act and the Health Act. The study has been registered at the Danish Data Protection Agency.

Recruitment and inclusion will take place as previously described (section 3.3.1). Participation requires a signed statement of consent. Patients can withdraw their participation consent and withdraw from the research project at any time without this having any effect on their right to current or future treatment. Furthermore, the patient is entitled to bring a bystander to the information interview and is entitled to reflection time before any declaration of consent is signed.

The important objective of the study is to investigate whether pro-active and *pre-emptive* treatment against COVID-19 can reduce the length of hospitalisation and the risk of intensive care and improve the survival of patients - an area that has so far been poorly researched and where the need for evidence-based guideline for handling and processing is large and very urgent.

Potential disadvantages and side effects are described in the separate section 5. Among other things, it appears that the likelihood of serious adverse reactions to both treatment and examinations is rare. In addition, the treating physician can always discontinue treatment if it is considered contraindicated.

Placebo is given patients allocated to the control group as no specific standard medical treatment is available.

The experimental method and statistical analyses have been carefully considered in order to be able to disseminate and apply relevant and secure research results to clinical practice.

Based on the above considerations, we believe that the experiment is ~~sound~~ ethically sound and can be conducted without exposing the test participant to unjustifiable risks.

12. Recruitment of subjects and informed consent

At each trial centre, screening of patients admitted with a positive SARS-CoV-2 test is performed. Patients are assessed against the inclusion and exclusion criteria of the attending physician who receives the patient's consent to contact the investigator. The Investigator then contacts the patient for recruitment to the study. Disclosure of information about the study and obtaining informed consent may also be undertaken by other healthcare professionals. This includes research assistants (medical students), clinical nursing specialists and project nurses (See below for specific requirements). These are all separately trained in the task and have the opportunity to call a physician should any medical issues arise. They can also contact the coordinating investigator as well as a hotline team for the trial should any questions arise about informed consent. This hotline is available 24 hours a day. All patients are offered a consultation with a physician if they ask for it. For project nurses, the following applies specifically: i) must have at least 5 years of seniority. These requirements are verified by primary investigator from each site that afterwards creates a document for these individuals from which the above specific requirements are verified. This document is dated and signed.

If a patient is considered suitable, the person will be invited to participate in the project. Participation in the trial is voluntary. Informed consent is obtained from the participants of the trial acc. to Executive Order No. 1149 of 30 September 2013 on information and consent for participation in health science research projects and on notification and supervision of health science research projects. The first contact with the the potential participant in the trial will be at admission to one of the participating departments. Participant information is provided both orally and in writing, and the patient is informed that they are entitled to 24 hours of reflection time before consent is given for participation in the trial. Participants who wish to do so themselves after the period of reflection time may give consent in connection with the information meeting.

The right to a bystander is ensured by the patient being able to bring a bystander, however, subject to COVID isolation rules. If no bystanders come to the first call, they are ensured afterwards to a bystander, when the patient is out of isolation. It is ensured that the conversations are undisturbed by using the patient's isolation room. If the doctor carries a "pager" or telephone, these are handed in prior to the call. The trial participant will be provided with the document "The research subject's rights in a health science research project", which contains information about confidentiality, access to documents and access to complaints. The subjects are protected under the Personal Data Processing Act. The trial has been reported to the Regional Science Ethics Committee, the Danish Medicines Agency and the Danish Data Protection Agency.

It must be ensured at all times that subjects have consented to participate in clinical trials. If an isolated subject with COVID-19 can sign consent declaration via electronic tool, this can be used instead of consent with signature. This can be, for example, a mobile phone, an iPad, a laptop with secure identification, for example by an easy ID (or other solution that meets the OCES standard). If the above described solution is not possible, the following solutions can be used as temporary documentation for the consent:

• Copy of signed consent declaration – e.g. using camera: The subject can sign the consent form as usual. Since the signed form must not leave the isolation room, the signature can be documented in the form of a photograph of the signed form, for example through a window.

• If the test subject cannot sign the consent declaration himself, e.g. due to problems with having electronic equipment in the room, or obtaining documentation for the consent out of the room, the witness can sign on behalf of the subject: If the subject verbally consents, a witness can on behalf of the subject sign the consent form. For both of the above solutions, documentation (photo and witness signature) will be filed in the investigator's section of the Trial Master File (TMF). Furthermore, it is ensured that the Data Protection Regulation and the Data Protection Act are complied with, although documentation of the consent is temporarily different than it usually is. If the situation is normalised, the correct signed consent form must be obtained from the subject as soon as possible.

13. Exclusion and interruption of trials:

Regular monitoring and quality control of the study will be carried out. If the physician responsible for the study deems it necessary, the physician may during treatment take the subject out of the study. The physician may also terminate the study at any time if there is a medical justification (such as the development of allergies to medicines), a safety risk or a requirement from the authorities. The test subject may also withdraw their informed consent and withdraw from the investigation at any time, as mentioned in the above paragraph.

14. Information on compensation and compensation schemes:

Patients who participate in these studies and who believe they have suffered injury can seek compensation through the patient compensation (http://patienterstatningen.dk/) cf. Danish law.

15. References:

1. Guan WJ, Ni ZY, Hu Y, Liang WH, Ou CQ, He JX, et al. Clinical Characteristics of Coronavirus Disease 2019 in China. N Engl J Med. 2020.

2. Zhou F, Yu T, Du R, Fan G, Liu Y, Liu Z, et al. Clinical course and risk factors for mortality of adult inpatients with COVID-19 in Wuhan, China: a retrospective cohort study. Lancet. 2020.

3. Mo P, Xing Y, Xiao Y, Deng L, Zhao Q, Wang H, et al. Clinical characteristics of refractory COVID-19 pneumonia in Wuhan, China. Clin Infect Dis. 2020.

4. Yang X, Yu Y, Xu J, Shu H, Xia J, Liu H, et al. Clinical course and outcomes of critically ill patients with SARS-CoV-2 pneumonia in Wuhan, China: a single-centered, retrospective, observational study. Lancet Respir Med. 2020.

5. Murthy S, Gomersall CD, Fowler RA. Care for Critically Ill Patients With COVID-19. JAMA. 2020.

6. Uzun S, Djamin RS, Kluytmans JA, Mulder PG, van't Veer NE, Ermens AA, et al. Azithromycin maintenance treatment in patients with frequent exacerbations of chronic obstructive pulmonary disease (COLUMBUS): a randomised, double-blind, placebo-controlled trial. Lancet Respir Med. 2014;2(5):361-8.

7. Albert RK, Connett J, Bailey WC, Casaburi R, Cooper JA, Jr., Criner GJ, et al. Azithromycin for prevention of exacerbations of COPD. N Engl J Med. 2011;365(8):689-98.

8. Gibson PG, Yang IA, Upham JW, Reynolds PN, Hodge S, James AL, et al. Effect of azithromycin on asthma exacerbations and quality of life in adults with persistent uncontrolled asthma (AMAZES): a randomised, double-blind, placebo-controlled trial. Lancet. 2017;390(10095):659-68.

9. Altenburg J, de Graaff CS, Stienstra Y, Sloos JH, van Haren EH, Koppers RJ, et al. Effect of azithromycin maintenance treatment on infectious exacerbations among patients with non-cystic fibrosis bronchiectasis: the BAT randomized controlled trial. JAMA. 2013;309(12):1251-9.

10. Segal LN, Clemente JC, Wu BG, Wikoff WR, Gao Z, Li Y, et al. Randomised, double-blind, placebo-controlled trial with azithromycin selects for anti-inflammatory microbial metabolites in the emphysematous lung. Thorax. 2017;72(1):13-22.

11. Kawamura K, Ichikado K, Takaki M, Eguchi Y, Anan K, Suga M. Adjunctive therapy with azithromycin for moderate and severe acute respiratory distress syndrome: a retrospective, propensity score-matching analysis of prospectively collected data at a single center. Int J Antimicrob Agents. 2018;51(6):918-24.

12. Beigelman A, Mikols CL, Gunsten SP, Cannon CL, Brody SL, Walter MJ. Azithromycin attenuates airway inflammation in a mouse model of viral bronchiolitis. Respir Res. 2010;11:90.

13. Gielen V, Johnston SL, Edwards MR. Azithromycin induces anti-viral responses in bronchial epithelial cells. Eur Respir J. 2010;36(3):646-54.

14. Li C, Zu S, Deng YQ, Li D, Parvatiyar K, Quanquin N, et al. Azithromycin Protects against Zika virus Infection by Upregulating virus-induced Type I and III Interferon Responses. Antimicrob Agents Chemother. 2019.

15. Savarino A, Boelaert JR, Cassone A, Majori G, Cauda R. Effects of chloroquine on viral infections: an old drug against today's diseases? Lancet Infect Dis. 2003;3(11):722-7.

16. Lee SJ, Silverman E, Bargman JM. The role of antimalarial agents in the treatment of SLE and lupus nephritis. Nat Rev Nephrol. 2011;7(12):718-29.

17. Vincent MJ, Bergeron E, Benjannet S, Erickson BR, Rollin PE, Ksiazek TG, et al. Chloroquine is a potent inhibitor of SARS coronavirus infection and spread. Virol J. 2005;2:69.

18. Colson P, Rolain JM, Raoult D. Chloroquine for the 2019 novel coronavirus SARS-CoV-2. Int J Antimicrob Agents. 2020;55(3):105923.

19. Gao J, Tian Z, Yang X. Breakthrough: Chloroquine phosphate has shown apparent efficacy in treatment of COVID-19 associated pneumonia in clinical studies. Biosci Trends. 2020;14(1):72-3.
